# Cross-sectional examination of characteristics of higher-dose buprenorphine prescriptions during the era of illicit fentanyl

**DOI:** 10.1186/s13722-025-00547-0

**Published:** 2025-04-09

**Authors:** Bradley D. Stein, Flora Sheng, Brendan K. Saloner, Adam J. Gordon, Jessica S. Merlin

**Affiliations:** 1 RAND, Pittsburgh, PA USA; 2 RAND, Arlington, VA USA; 3 Johns Hopkins Bloomberg School of Public Health, Baltimore, MD USA; 4 Program for Addiction Research, Clinical Care, Knowledge and Advocacy, Division of Epidemiology, Department of Internal Medicine, University of Utah School of Medicine, Salt Lake City, UT USA; 5 Informatics, Decision-Enhancement, and Analytic Sciences Center, VA Salt Lake City Health Care System, Salt Lake City, Utah USA; 6 Department of Medicine, University of Pittsburgh School of Medicine, Pittsburgh, PA USA

**Keywords:** Opioid use disorder, Buprenorphine, Insurance

## Abstract

**Background:**

In response to greater illicit fentanyl use, buprenorphine daily doses exceeding the FDA’s recommended target daily dose (16 mg) and maximum suggested daily dose (24 mg) may provide better outcomes, but little is known about higher dosage prescribing patterns. To better understand buprenorphine prescribing patterns, this manuscript examines the frequency and characteristics of dispensed buprenorphine of ≤ 16mg, > 16-24 mg, and > 24 mg daily dose.

**Methods:**

We used IQVIA data to conduct a cross-sectional study of opioid use disorder-indicated buprenorphine prescriptions dispensed at retail pharmacies January 2019 - December 2020; categorized prescriptions as ≤ 16mg, > 16 to 24 mg, and > 24 mg daily dose; and examined overall rates and rates by patient, insurer and county characteristics, and prescriber specialty. We categorized buprenorphine prescriptions by patient sex, age cohort, primary payment source, and prescriber specialty and state and conducted univariate and bivariate analyses of buprenorphine daily dose categories overall and among clinicians frequently prescribing buprenorphine at the highest doses, > 24 mg.

**Results:**

Approximately 19.5% (*n* = 5,568,964) of the 28 million buprenorphine prescriptions from 68,898 clinicians were > 16-24 mg; 2% (*n* = 641,390) were > 24 mg. Approximately 26% (*n* = 17,939) of clinicians wrote at least one prescription > 24 mg; 2,780 clinicians (4% of buprenorphine prescribers) were responsible for 82.2% (*n* = 527,597) of dispensed prescriptions > 24 mg. 28% of prescriptions > 24 mg written by these prescribers were cash-pay, 12.5% covered by Medicaid, and 6.7% covered by Medicare. There was no correlation between state fentanyl overdose rate and buprenorphine prescriptions > 24 mg per 1,000,000 residents.

**Conclusions:**

In 2019–2020, fewer than 3% of dispensed buprenorphine prescriptions exceeded the FDA suggested maximum of 24 mg daily dose; 80% of the prescriptions for a > 24 mg daily dose were written by 4% of buprenorphine prescribers. As clinicians and policymakers pay greater attention to ensuring individuals are receiving buprenorphine dosages adequate to effectively treat their opioid use disorder, the recently revised FDA recommendations may encourage such behavior. Additionally, disproportionate reliance on cash payment for higher daily doses suggests public and private insurers could facilitate access to such treatment when appropriate.

**Supplementary information:**

The online version contains supplementary material available at 10.1186/s13722-025-00547-0.

## Background

As illicitly manufactured fentanyl fuels an increasingly potent drug supply [1], medication treatment of opioid use disorder (OUD) may need to evolve [2], including clinicians’ approach to buprenorphine prescribing, a medication used to treat OUD that can decrease overdose deaths and adverse healthcare outcomes [3, 4]. The majority of individuals receive the FDA-recommended target daily dose of 16 mg or less [5, 6], but fentanyl can induce greater tolerance than many other opioids [7], requiring higher daily buprenorphine doses [8–11] to adequately control cravings [12]. Observational studies have found an association between buprenorphine daily doses above both 16 mg and above the maximum indicated daily dose (24 mg), and outcomes including longer retention in medication treatment for opioid use disorder, longer periods before relapse [6, 13–17], and lower rates of emergency department and inpatient use [18]. Clinicians are able to prescribe doses above the suggested limit, but such prescribing is often considered “off-label,” and the FDA labeling has contributed to both states and insurers implementing limits for higher daily doses that may be inconsistent with federal limits and emerging research.

Uncertainty about the therapeutic value of buprenorphine at higher doses may be due to a recognition that mu-opioid receptor occupancy may become full at lower doses [19–21]. However, buprenorphine has relatively minor safety risks at higher doses compared to full agonist medications. There are recent anecdotal reports of greater use of buprenorphine doses > 24 mg daily [22]. However, little is known about patterns of high-dose buprenorphine prescribing. To address these knowledge gaps, we used national pharmacy claims data to examine: (1) frequency and characteristics of dispensed buprenorphine prescriptions where the daily dose was > 24 mg and > 16–24 mg, (2) among these prescriptions, variation in patient, insurer, and prescriber characteristics, and 3), state variation in the dispensing of high-dose buprenorphine prescriptions > 24 mg.

## Methods

We identified buprenorphine formulations indicated for OUD dispensed between January 2019 and December 2020 using IQVIA Real World Data – Longitudinal Prescriptions [23], which capture approximately 90% of all prescriptions filled at U.S. retail pharmacies.

We categorized all dispensed buprenorphine prescriptions as ≤ 16mg, > 16-24 mg, and > 24 mg daily dose, considering the dose in any sublingual formulations dispensed the same day as cumulative. We chose these categories to reflect dispensed prescriptions greater than the FDA recommended daily target dose (16 mg daily) and maximum suggested dose (24 mg daily) [24]. To better understand patterns of buprenorphine prescribing, we calculated the rate of > 24 mg and > 16-24 mg dispensed buprenorphine prescriptions overall and by prescriber, patient, and county characteristics. Using information on clinician specialty included in the IQVIA data generated from the American Medical Association (AMA) Masterfile and other data sources using clinician NPI and refined using the NPPES, we categorized prescribers by specialty/provider type: addiction specialists (including both addiction medicine and addiction psychiatry); adult primary care physicians (including internal medicine and family practice); advance practice providers (APP; primarily nurse practitioners and physician assistants); psychiatrists; pain specialists; emergency department physicians; and other physicians.

We categorized prescriptions by patient sex, age cohort (12–17 years, 18–25, 26–35, 36–45, 46–55, 56–65 and over 65 years), and primary source of payment (Medicaid; Medicare; commercial insurance; cash payment; prescription discount cards/coupons/vouchers; or other, which included Tricare and workers compensation). We used the 5-digit Federal Information Processing Standards (FIPS) code of the prescriber to determine the prescriber’s state; we used the 2019 restricted multiple-cause-of-death mortality file from the Centers for Disease Control and Prevention National Vital Statistics System [1], corresponding to the time frame of pharmacy claims, to determine fatal overdoses involving fentanyl per 1,000,000 state residents.

### Analysis

We conducted univariate and bivariate analyses of dispensed buprenorphine daily dose categories for 2019–2020. Supplement Tables 1 and 2 provide this information for each year separately. Given that 82% of dispensed buprenorphine prescriptions > 24 mg written by 4% (*n* = 2780) of clinicians who prescribed any dispensed buprenorphine > 24 mg (hereafter frequent high-dose prescribers), we compared prescriptions > 24 mg written by the frequent high dose prescribers to those written by prescribers who were not frequent high dose prescribers. We also examined the correlation between states’ buprenorphine dispensing rate for buprenorphine doses > 24 mg and the 2018 rate of fatal overdoses involving fentanyl (hereafter fentanyl fatal overdose rate) using Pearson correlation coefficients. Analyses were conducted using SAS version 9.4.

## Results

**Fig. 1:**
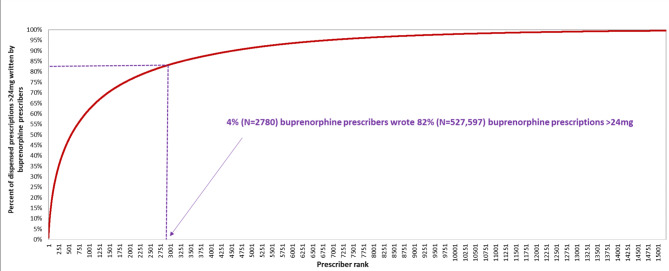
Cumulative percentage of dispensed buprenorephine prescriptions > 24 mg written by prescribers of > 24 mg buprenorphine

In 2019–2020, we identified over 28 million dispensed buprenorphine prescriptions written by 68,898 clinicians, of which approximately 19.5% (*n* = 5,568,964) were > 16-24 mg and 2.2% (*n* = 641,930) were > 24 mg (Table 1). Approximately 26% (*n* = 17,939) of clinicians had written a dispensed buprenorphine prescription with a daily dose > 24 mg at least once. Having written buprenorphine prescriptions with a daily dispensed dose of > 24 mg is highly concentrated (Fig. 1); 2780 clinicians (hereafter frequent high-dose prescribers) comprising 4% of all active buprenorphine prescribers. The median number of buprenorphine prescriptions > 24 mg written by such frequent high-dose prescribers in 2019–2020 was 96 (IQR 58 to 188), and they were responsible for 82.2% (*n* = 527,597) of all dispensed prescriptions > 24 mg. The median number of dispensed buprenorphine prescriptions > 24 mg among the remaining 15,159 clinicians who were not frequent high dose prescribers was 3 (IQR 1 to 10).

**Table 1:** Patient, payer, and clinician specialty of dispensed buprenorphine prescriptions

	> 24 mg daily dose	> 16 and ≤ 24 mg daily dose	16 mg or less daily dose
Total	641,930	5,568,964	22,375,122
Payer	%	%	%
Commercial insurance	17.6	23.3	23.9
Self-pay	25.7	10.1	6.8
Medicaid	14.3	34.4	40.7
Medicare	7.7	11.4	8.3
Discount card/voucher	24.7	12.3	11.5
Other	10.0	8.5	8.7
Specialty			
Addiction specialist	4.9	3.3	2.5
Emergency physician	2.2	2.0	2.1
Psychiatrist	14.4	12.1	11.0
Other	5.4	5.1	5.0
APP	13.7	22.1	24.7
Primary Care Physician	51.9	48.6	48.6
Pain specialist	7.7	6.8	6.1
Age			
12–17	0.1	0.1	0.1
18–25	2.4	3.1	4.3
26–35	30.9	31.5	33.9
36–45	34.2	32.5	32.7
46–55	18.0	17.9	16.2
56–65	11.2	11.5	9.7
66+	3.2	3.4	3.1
Sex			
Male	58.7	55.7	54.4
Female	41.3	44.3	45.6

Medicaid was the payer for 40.7% of dispensed buprenorphine ≤ 16 mg and 34.4% of > 16-24 mg prescriptions but only 14.3% of prescriptions > 24 mg, with commercial insurance responsible for 23.9%, 23.3%, and 17.6% respectively (Table 1). In contrast, 6.8% of the lowest dose category was cash pay, compared to 10.1% of > 16-24 mg prescriptions and 25.7% of prescriptions > 24 mg; discount card/vouchers followed a similar pattern, comprising 11.5% <16 mg, compared to 12.3% of > 16-24 mg prescriptions and 24.7% of prescriptions > 24 mg.

Comparing buprenorphine prescriptions for > 24 mg written by frequent high-dose prescribers to those written by other prescribers (Table 2), we found that 28% of prescriptions > 24 mg written by frequent high-dose prescribers were cash pay, almost double the 15% written by other prescribers (*p* < 0.01). Similarly, the 12.5% of dispensed prescriptions > 24 mg written by frequent high-dose prescribers paid for by Medicaid and the 6.7% paid for by Medicare were many fewer than the 23.0% Medicaid and 11.9% Medicare written by other prescribers, respectively(*p* < 0.01).

**Table 2:** Buprenorphine prescriptions > 24 mg by frequent prescribers of high-dose buprenorphine and other prescribers

	Frequent high-dose prescribers (*n* = 2780)	Not frequent high-dose prescribers (*n* = 66118)
	> 24 mg dose buprenorphine (*n* = 114,333)	> 24 mg dose buprenorphine (*n* = 527,597)
	%	%
Payer		
Commercial insurance	20.0	17.1
Self-pay	15.0	28.0
Medicaid	23.0	12.5
Medicare	11.9	6.7
Discount card/voucher	20.1	25.7
Other	9.9	10.0
Specialty		
Addiction specialist	2.0	5.5
Other	4.0	5.7
Emergency physician	1.6	2.3
Psychiatrist	12.3	14.8
APP (advance practice providers)	28.4	10.5
Primary Care Physician	42.7	53.8
Pain specialist	9.0	7.4
Age		
12–17	0.1	0.1
18–25	3.1	2.3
26–35	27.8	31.5
36–45	30.8	35.0
46–55	19.5	17.7
56–65	13.9	10.6
66+	4.8	2.8
Sex		
Male	56.4	59.2
Female	43.6	40.8

Buprenorphine prescribing of > 24 mg varied by specialty: adult PCPs were responsible for having written more than half (51.9%) of all dispensed prescriptions > 24 mg, slightly more than the 48.6% of lower dose prescriptions they wrote (*p* < 0.001). Among all PCPs having written at least one dispensed prescription > 24 mg, 5% were frequent high-dose prescribers. Psychiatrists (14.4%), APPs (13.7%), and pain specialists (7.7%) were responsible for the next largest number of prescriptions > 24 mg. Similar to PCPs, both psychiatrists and pain specialists were responsible for a slightly greater percentage of prescriptions > 24 mg compared to prescriptions of > 16-24 mg or ≤ 16 mg (*p* < 0.001 for both). Among all psychiatrists and pain specialists having written at least one dispensed prescription > 24 mg, 5% of both psychiatrists and pain specialists were frequent high-dose prescribers.

In contrast, APPs were responsible for only 13.7% of prescriptions > 24 mg; they wrote a much larger percentage of > 16-24 mg (22.1%) and ≤ 16 mg (24.7%) prescriptions (*p* < 0.001), and only 2% of APPs having written any high-dose prescriptions were frequent high-dose prescribers. Addiction specialists were responsible for only 4.9% of all buprenorphine prescriptions > 24 mg, although this is greater than the 3.3% of > 16-24 mg and 2.5% of ≤ 16 mg prescriptions they wrote (*p* < 0.001), and 14% of addiction specialists who wrote at least one high-dose prescription were frequent high-volume prescribers. Differences in buprenorphine prescriptions > 24 mg by patient age and sex were statistically significant but relatively modest.

The study was approved with a waiver of consent by the corresponding author’s IRB.

### State variation in rate of prescribing of buprenorphine > 24 mg

**Fig. 2:**
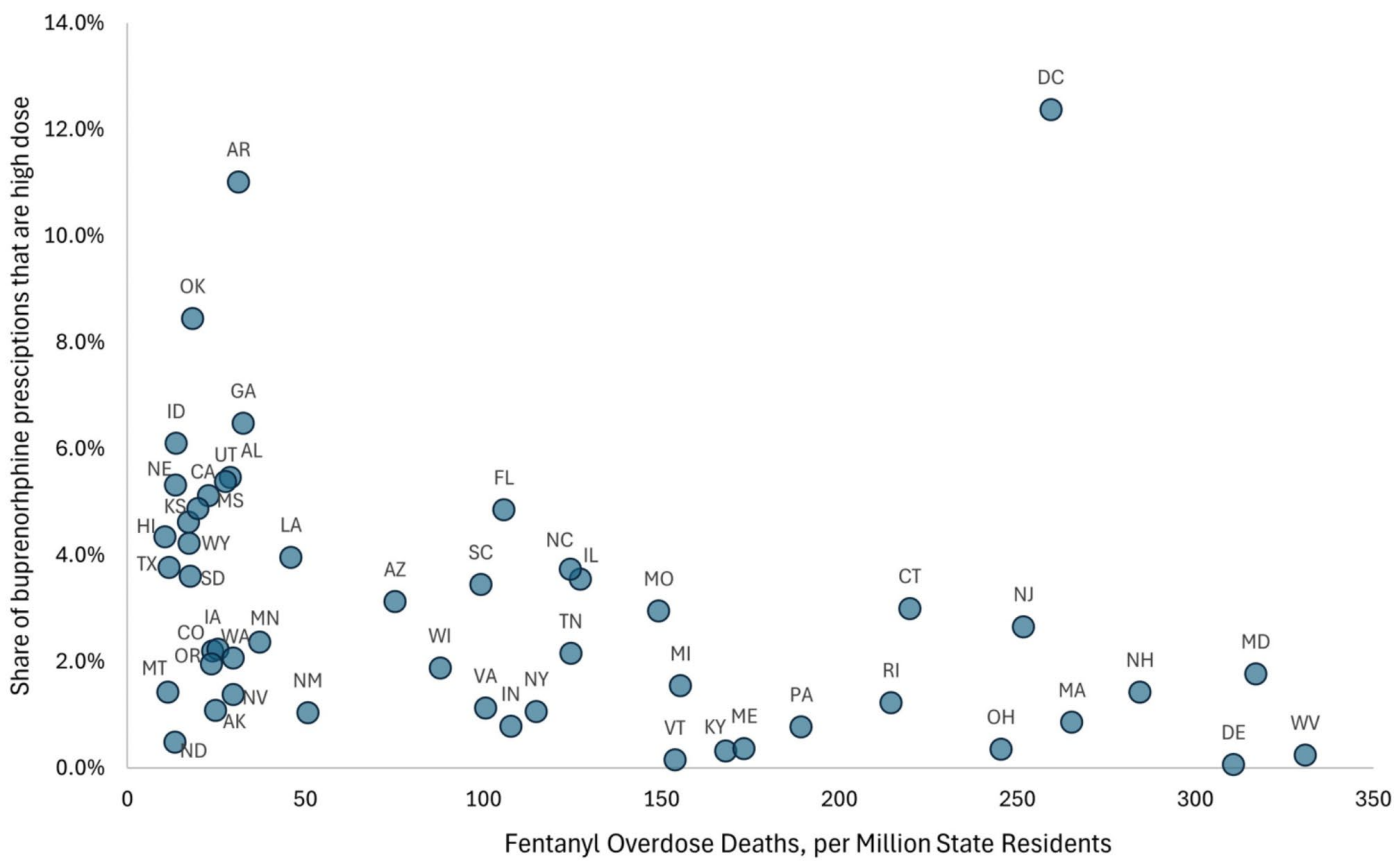
State high dose buprenorphine dispensing and 2018 state fentanyl fatal overdose rate Pearson Correlation ρ= -0.31 (*p* = 0.026)

**Fig. 3:**
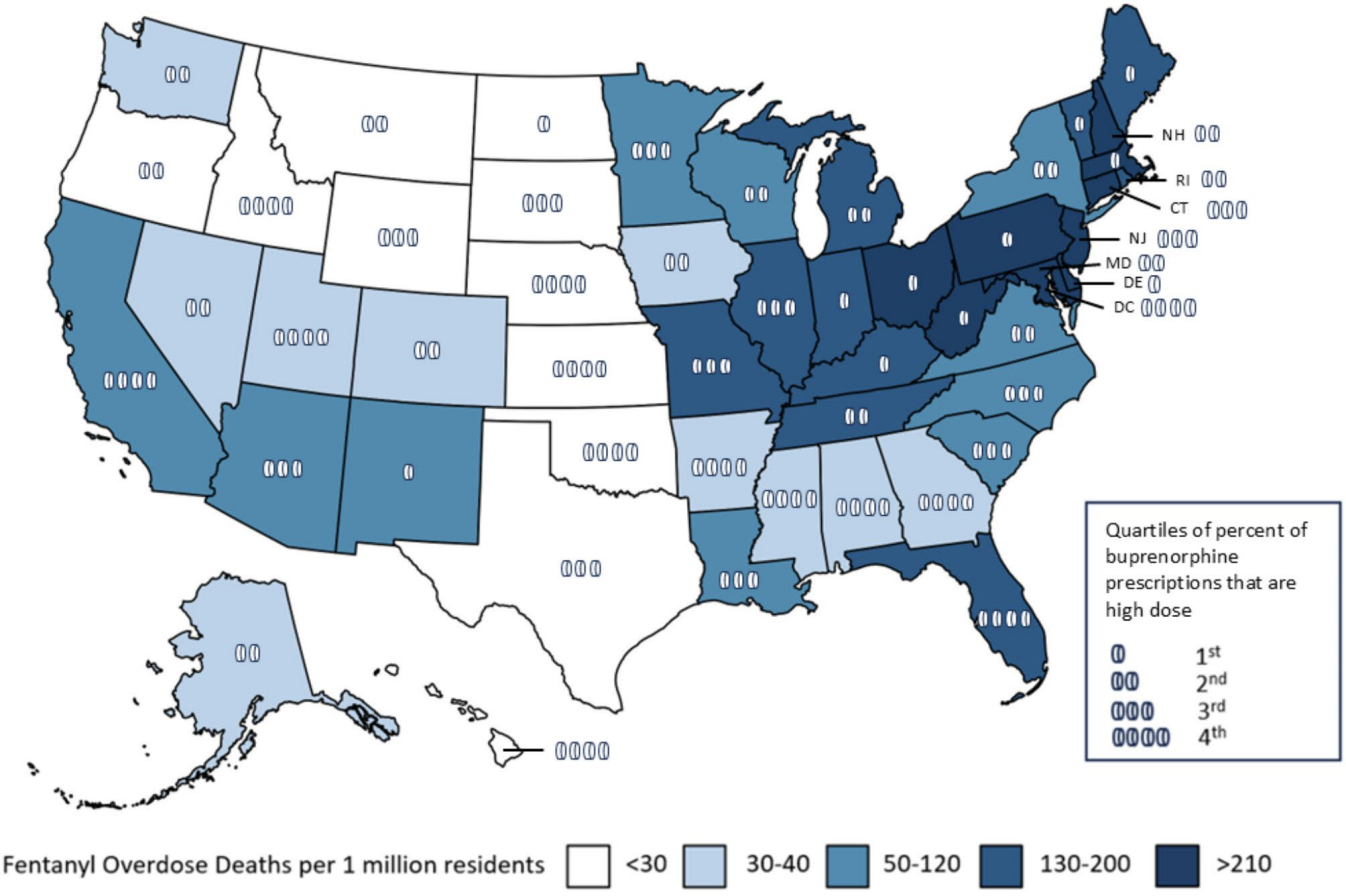
State high dose buprenorphine dispensing and fentanyl fatal overdose rate

There was also substantial variation in the percentage of dispensed buprenorphine prescriptions that were > 24 mg, ranging from greater than 7% of all buprenorphine prescriptions in Arkansas and Oklahoma to less than 0.5% in Delaware, Maine, Ohio, and West Virginia. As Fig. 2 illustrates, percentage of dispensed buprenorphine prescriptions that were > 24 mg was negative correlated (Pearson Correlation ρ = -0.31, *p* = 0.026) with the 2018 fatal fentanyl overdose rate. The rate of buprenorphine prescriptions > 24 mg dispensed also varied substantially across states, ranging from 4,642 per 1,000,000 residents in Alabama to 42 per 1,000,000 residents in Delaware, with an average across states of 1,207 per 1,000,000 residents. However, the rate of buprenorphine prescribing > 24 mg per 1,000,000 residents was not significantly correlated (Rho = 0.063; *p* = 0.66) with 2018 fatal fentanyl overdose rate (Supplement Fig. 1). Figure 3 illustrates some of the regional patterns, with several states with high fentanyl overdose rates, such as Ohio, Pennsylvania, Massachusetts, and West Virginia, being in the lowest quartile of the percent of buprenorphine prescriptions that were > 24 mg, while states such as Oklahoma, Kansas, Nebraska, and Idaho, which were in the highest quartile of the percent of buprenorphine prescriptions that were > 24 mg were among the states with the lowest fatal fentanyl overdose rates. Supplement Fig. 2 illustrates > 24 mg buprenorphine prescribing rates per 1,000,000 state residents.

## Discussion

In an era where fentanyl is nearly ubiquitous in the illicit opioid supply, higher doses of buprenorphine may become increasingly important. The FDA guideline has suggested that buprenorphine should only be prescribed at 24 mg or less per day, but the possible therapeutic value of higher doses of buprenorphine resulted in a December 2024 recommendation to revise the indication [25]. Our analysis of 2019-20 national pharmacy claims data suggests that prescribing of buprenorphine > 24 mg/day has been relatively rare, occurring in only 2.5% of all prescription fills; fewer than 20% of dispensed doses are in the > 16 mg to 24 mg daily dose range.

Prior research has shown that relatively few clinicians are responsible for the majority of buprenorphine prescribing overall [26]. We find substantial concentration among prescribers of buprenorphine doses > 24 mg: fewer than 3000 clinicians were responsible for over 80% of > 24 mg buprenorphine prescribing. Buprenorphine prescribing clinicians have expressed concerns about regulatory surveillance and diversion of prescribed buprenorphine [27, 28], and it is possible that such concerns might diminish willingness to prescribe higher doses of buprenorphine. In light of the FDA recommendation of revised guidelines regarding buprenorphine doses > 24 mg, efforts to address these concerns may be helpful in facilitating greater use of such doses.

We also find a much higher rate of cash pay for buprenorphine prescriptions > 24 mg than among all prescriptions. Some of this may reflect insurer dosage limit policies, which often limit buprenorphine doses to 24 mg or 16 mg or less daily [29]. However, some of the difference may also stem from the practice patterns of clinicians who frequently prescribe buprenorphine doses > 24 mg, as their cash pay rates are higher than rates of other clinicians for dispensed prescriptions > 24 mg. High patient volume buprenorphine prescribers have higher rates of cash pay buprenorphine than other prescribers [30], and it may be that such prescribers comprise a larger percentage of more frequent prescribers of buprenorphine doses > 24 mg.

Cash payment for higher doses of buprenorphine may have equity implications for socioeconomically disadvantaged individuals who might benefit from higher dosages. In February 2024, a typical daily cash price for 24 mg of Suboxone would be about $27 (and roughly half the price for generic, when a coupon is applied) [31]. The financial strain of cash payment may also increase buprenorphine diversion as individuals may look to sell excess medication to pay for their prescription [32] and may also contribute to undertreatment of OUD. As findings continue to emerge that higher doses may be beneficial [6, 13–16, 18], it will be important to ensure that higher-dose buprenorphine is readily available to individuals without commercial insurance.

Although exposure to more potent opioids is a common reason provided for prescribing of higher doses of buprenorphine, we find that many of the states with prescribers who are writing the highest rate of buprenorphine prescriptions > 24 mg dispensed per capita, such as Idaho and Oklahoma, had among the lowest fentanyl-related overdose rates in the preceding year. In contrast, several of the states with the highest fentanyl overdose rate, such as Ohio and Vermont, have among the lowest rate of buprenorphine prescriptions > 24 mg. Many factors can influence clinicians’ buprenorphine prescribing patterns, including state and insurer policies [33, 34], and potentially local access to opioid treatment programs providing methadone, which can also effectively treat individuals using higher potency opioids. While many state Medicaid agencies and other insurers have long had guidelines limiting buprenorphine prescription daily dose, some states in recent years have begun to relax those limits, allowing doses up to 32mg [35], and policies such as continuing medical education requirements and prior authorization policies have been shown to influence buprenorphine dispensing [36–38], and additional research is needed to better understand the relationship between buprenorphine prescribing > 24 mg in real world settings, fentanyl-related overdose rates, and state policies.

Our findings must be considered within the context of the study limitations. Our buprenorphine prescribing data are from a period where fentanyl was already widespread in eastern states but had not yet become as widespread in western states [39]. We do not know to what extent prescribing patterns may have changed as the opioid crisis has evolved, and fentanyl related deaths have continued to increase and become more common in western states. Nor do we know how the response to the COVID pandemic in 2020 may have changed buprenorphine dose prescribing patterns and influenced our findings. We also do not have accurate information on OUD rates, and we are unable to assess if there is a correlation between rates of higher buprenorphine use and OUD rates. We only observe the primary form of payment recorded by the pharmacy and have no information on secondary forms of payment, such as secondary insurers or some form of insurance secondary to use of a discount card/voucher. We have no information on patient clinical status or diagnosis, so we do not know to what extent dispensed buprenorphine indicated for OUD is being used to manage pain. We also cannot systematically observe long-acting buprenorphine formulations, as these appear in both service claims (which we do not observe) as well as pharmacy claims, and we cannot determine how many individuals are receiving buprenorphine doses > 16 mg or > 24 mg through a combination of long-acting buprenorphine and dispensed buprenorphine. Nor can we determine if individuals dispensed multiple overlapping buprenorphine prescriptions are taking them in a way that results in a daily dose above 16 mg or 24 mg. As a result, our rates of buprenorphine prescribing > 16–24 and > 24 mg may be underestimated. We also have no information on quality of care or treatment outcomes; nor do we know if individuals are taking buprenorphine prescriptions > 16-24 mg or > 24 mg as prescribed, information very much needed by the field.

As clinicians and policymakers [29, 35, 40] consider the potential benefits of buprenorphine > 16 mg and > 24 mg in response to the fentanyl crisis, this descriptive study using national pharmacy data contributes substantial new information. As only about 2% of prescriptions are for buprenorphine doses > 24 mg, a better understanding is needed of the size and characteristics of the population that could potentially benefit and to ensure that such individuals can receive the most clinically beneficial dosages. The disproportionate reliance on cash payment and discount cards for higher dose buprenorphine prescriptions suggests that public and private insurers could play a greater role in facilitating access to such treatment when appropriate. A concerted focus on updating dosage guidelines, coupled with supportive policies, may be needed to help support the national goal of reducing overdose deaths and increasing recovery.

## Electronic supplementary material

Below is the link to the electronic supplementary material.

Supplementary Material 1

## Data Availability

Data is not publicly available due to a restricted Data Use Agreement with IQVIA.

## Abbreviations

AMA: American Medical Association
APP: Advanced practice providers
FDA: Food and Drug Administration
FIPS: Federal Information Processing Standards
IQR: Interquartile range
NPI: National Provider Identifier
NPPES: National Plan & Provider Enumeration System
OUD: Opioid use disorder
