# Supplementary material for: Cross-sectional examination of characteristics of higher-dose buprenorphine prescriptions during the era of illicit fentanyl
Source: Addict Sci Clin Pract. 2025 Apr 9;20:33. doi: 10.1186/s13722-025-00547-0 (PMC11980160; doi:10.1186/s13722-025-00547-0)

Supplement Table 1: Patient, payer, and clinician specialty of dispensed buprenorphine prescriptions in 2019 and 2020

|  | 2019 (N=14,098,970) | | | | | | | 2020 (N=14,487,046) | | | | | | |
| --- | --- | --- | --- | --- | --- | --- | --- | --- | --- | --- | --- | --- | --- | --- |
| **Characteristics** | High dose (>24mg) | | Non-high dose BUP Rx (16-24] | | Non-high dose BUP Rx (<=16) | | Chi-Square p-value | High dose(>24mg) | | Non-high dose BUP Rx (16-24] | | Non-high dose BUP Rx (<=16) | | Chi-Square p-value |
|  | N | col % | N | col % | N | col % |  | N | col % | N | col % | N | col % |  |
| **Total** | 350,047 | 100.0% | 2,681,442 | 100.0% | 11,067,481 | 100.0% |  | 291,883 | 100.0% | 2,887,522 | 100.0% | 11,307,641 | 100.0% |  |
| **Primary payer for prescription** |  |  |  |  |  |  | <.0001 |  |  |  |  |  |  | <.0001 |
| Commercial | 57818 | 16.5% | 600863 | 22.4% | 2526177 | 22.8% |  | 55375 | 19.0% | 699289 | 24.2% | 2827666 | 25.0% |  |
| Cash | 92403 | 26.4% | 305109 | 11.4% | 840470 | 7.6% |  | 72365 | 24.8% | 257981 | 8.9% | 688106 | 6.1% |  |
| Medicaid | 46986 | 13.4% | 876461 | 32.7% | 4435795 | 40.1% |  | 45019 | 15.4% | 1040551 | 36.0% | 4674266 | 41.3% |  |
| Medicare | 24479 | 7.0% | 294883 | 11.0% | 885149 | 8.0% |  | 24737 | 8.5% | 339330 | 11.8% | 973897 | 8.6% |  |
| Discount Card | 91096 | 26.0% | 354753 | 13.2% | 1334210 | 12.1% |  | 67649 | 23.2% | 327649 | 11.3% | 1232849 | 10.9% |  |
| Other | 37265 | 10.6% | 249373 | 9.3% | 1045680 | 9.4% |  | 26738 | 9.2% | 222722 | 7.7% | 910857 | 8.1% |  |
| **Specialty** |  |  |  |  |  |  | <.0001 |  |  |  |  |  |  | <.0001 |
| Addiction | 17103 | 4.9% | 94169 | 3.5% | 307248 | 2.8% |  | 14184 | 4.9% | 87666 | 3.0% | 257175 | 2.3% |  |
| ED doctor | 8130 | 2.3% | 57435 | 2.1% | 243431 | 2.2% |  | 5920 | 2.0% | 53724 | 1.9% | 218327 | 1.9% |  |
| Psychiatrist | 53513 | 15.3% | 359552 | 13.4% | 1337703 | 12.1% |  | 38835 | 13.3% | 314626 | 10.9% | 1131352 | 10.0% |  |
| Other | 18394 | 5.3% | 145602 | 5.4% | 585814 | 5.3% |  | 16042 | 5.5% | 139650 | 4.8% | 523690 | 4.6% |  |
| APP | 40287 | 11.5% | 455412 | 17.0% | 2120502 | 19.2% |  | 47480 | 16.3% | 772918 | 26.8% | 3403126 | 30.1% |  |
| PCP | 185336 | 52.9% | 1373484 | 51.2% | 5753031 | 52.0% |  | 147521 | 50.5% | 1334941 | 46.2% | 5130972 | 45.4% |  |
| Pain | 27284 | 7.8% | 195788 | 7.3% | 719752 | 6.5% |  | 21901 | 7.5% | 183997 | 6.4% | 642999 | 5.7% |  |
| **Age** |  |  |  |  |  |  | <.0001 |  |  |  |  |  |  | <.0001 |
| 12-17 yo | 449 | 0.1% | 2937 | 0.1% | 10835 | 0.1% |  | 297 | 0.1% | 2773 | 0.1% | 12044 | 0.1% |  |
| 18-25 yo | 9503 | 2.7% | 92940 | 3.5% | 515253 | 4.7% |  | 6160 | 2.1% | 81204 | 2.8% | 447787 | 4.0% |  |
| 26-35 yo | 114972 | 32.8% | 874000 | 32.6% | 3871199 | 35.0% |  | 83135 | 28.5% | 880194 | 30.5% | 3717658 | 32.9% |  |
| 36-45 yo | 117560 | 33.6% | 858716 | 32.0% | 3548714 | 32.1% |  | 102054 | 35.0% | 951526 | 33.0% | 3769176 | 33.3% |  |
| 46-55 yo | 61111 | 17.5% | 472259 | 17.6% | 1772764 | 16.0% |  | 54487 | 18.7% | 524653 | 18.2% | 1848929 | 16.4% |  |
| 56-65 yo | 36740 | 10.5% | 298067 | 11.1% | 1034796 | 9.3% |  | 35057 | 12.0% | 342175 | 11.9% | 1132590 | 10.0% |  |
| 66+ yo | 9712 | 2.8% | 82523 | 3.1% | 313920 | 2.8% |  | 10693 | 3.7% | 104997 | 3.6% | 379457 | 3.4% |  |
| **Sex** |  |  |  |  |  |  | <.0001 |  |  |  |  |  |  | <.0001 |
| M | 204980 | 58.6% | 1494115 | 55.7% | 6007087 | 54.3% |  | 171866 | 58.9% | 1608227 | 55.7% | 6164263 | 54.5% |  |
| F | 145067 | 41.4% | 1187327 | 44.3% | 5060394 | 45.7% |  | 120017 | 41.1% | 1279295 | 44.3% | 5143378 | 45.5% |  |

Supplement Table 2. Buprenorphine prescriptions >24mg by frequent and other prescribers of high-dose buprenorphine in 2019 and 2020

|  | High dose BUP Rx by 50,531 High-dose providers in 2019 | | | | | High dose BUP Rx by 53,912 High-dose providers in 2020 | | | | |
| --- | --- | --- | --- | --- | --- | --- | --- | --- | --- | --- |
|  | >24mg BUP by 2,752 frequent high-dose provider | | >24mg BUP by 47,779 non-frequent high-dose provider | | Chi-square p value | >24mg BUP by 2,756 frequent high-dose provider | | >24mg BUP by 51,156 non-frequent high-dose provide | | Chi-square p value |
|  | N | col % | N | col % |  | N | col % | N | col % |  |
| **Total** | 59,847 | 100.0% | 290,200 | 100.0% |  | 54,486 | 100.0% | 237,397 | 100.0% |  |
| **Primary payer for prescription** |  |  |  |  | <.0001 |  |  |  |  | <.0001 |
| Commercial | 11500 | 19.2% | 46318 | 16.0% |  | 11352 | 20.8% | 44023 | 18.5% |  |
| Cash | 9709 | 16.2% | 82694 | 28.5% |  | 7496 | 13.8% | 64869 | 27.3% |  |
| Medicaid | 12980 | 21.7% | 34006 | 11.7% |  | 13327 | 24.5% | 31692 | 13.3% |  |
| Medicare | 6656 | 11.1% | 17823 | 6.1% |  | 6981 | 12.8% | 17756 | 7.5% |  |
| Discount Card | 12738 | 21.3% | 78358 | 27.0% |  | 10228 | 18.8% | 57421 | 24.2% |  |
| Other | 6264 | 10.5% | 31001 | 10.7% |  | 5102 | 9.4% | 21636 | 9.1% |  |
| **Specialty** |  |  |  |  | <.0001 |  |  |  |  | <.0001 |
| Addiction | 1338 | 2.2% | 15765 | 5.4% |  | 918 | 1.7% | 13266 | 5.6% |  |
| Other | 2500 | 4.2% | 15894 | 5.5% |  | 2091 | 3.8% | 13951 | 5.9% |  |
| ED doctor | 915 | 1.5% | 7215 | 2.5% |  | 859 | 1.6% | 5061 | 2.1% |  |
| Psychiatrist | 8214 | 13.7% | 45299 | 15.6% |  | 5901 | 10.8% | 32934 | 13.9% |  |
| APP | 14849 | 24.8% | 25438 | 8.8% |  | 17652 | 32.4% | 29828 | 12.6% |  |
| PCP | 26244 | 43.9% | 159092 | 54.8% |  | 22526 | 41.3% | 124995 | 52.7% |  |
| Pain | 5787 | 9.7% | 21497 | 7.4% |  | 4539 | 8.3% | 17362 | 7.3% |  |
| **Age** |  |  |  |  | <.0001 |  |  |  |  | <.0001 |
| 12-17 yo | 35 | 0.1% | 414 | 0.1% |  | 42 | 0.1% | 255 | 0.1% |  |
| 18-25 yo | 2003 | 3.3% | 7500 | 2.6% |  | 1525 | 2.8% | 4635 | 2.0% |  |
| 26-35 yo | 17625 | 29.5% | 97347 | 33.5% |  | 14180 | 26.0% | 68955 | 29.0% |  |
| 36-45 yo | 18032 | 30.1% | 99528 | 34.3% |  | 17144 | 31.5% | 84910 | 35.8% |  |
| 46-55 yo | 11651 | 19.5% | 49460 | 17.0% |  | 10690 | 19.6% | 43797 | 18.4% |  |
| 56-65 yo | 7878 | 13.2% | 28862 | 9.9% |  | 8000 | 14.7% | 27057 | 11.4% |  |
| 66+ yo | 2623 | 4.4% | 7089 | 2.4% |  | 2905 | 5.3% | 7788 | 3.3% |  |
| **Sex** |  |  |  |  | <.0001 |  |  |  |  | <.0001 |
| F | 26228 | 43.8% | 118839 | 41.0% |  | 23642 | 43.4% | 96375 | 40.6% |  |
| M | 33619 | 56.2% | 171361 | 59.0% |  | 30844 | 56.6% | 141022 | 59.4% |  |

Supplement Figure 1. State High Dose Buprenorphine Dispensing and 2018 State Fentanyl Fatal Overdose Rate


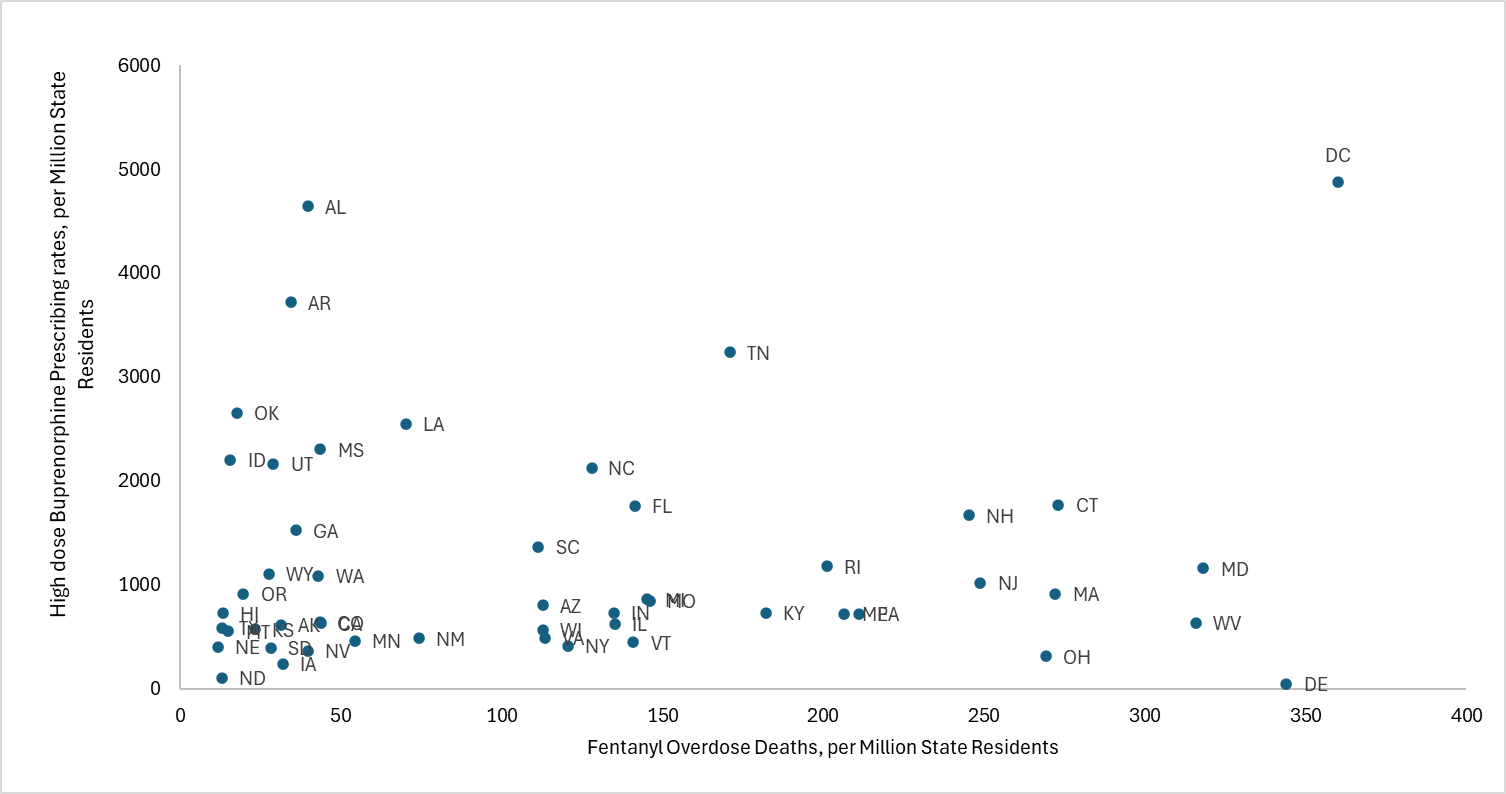


Rho=0.063; p=0.66

Supplement Figure 21 State High Dose Buprenorphine Dispensing and Fentanyl Fatal Overdose Rate


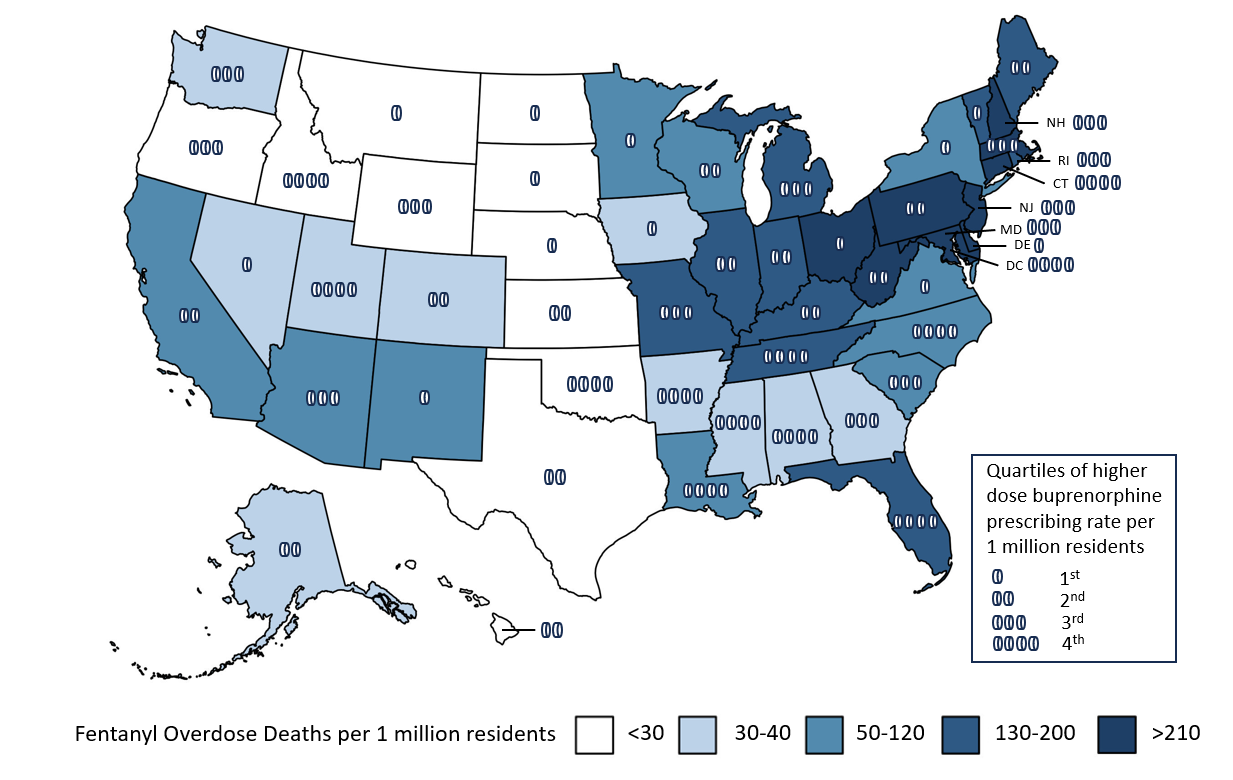

Supplement: Supplementary file 1 — Supplementary Material 1 [file 13722_2025_547_MOESM1_ESM.docx]
